# The proactive brain and the fate of dead hypotheses

**DOI:** 10.3389/fncom.2014.00138

**Published:** 2014-11-04

**Authors:** Amir Tal, Moshe Bar

**Affiliations:** The Leslie and Susan Gonda Multidisciplinary Brain Research Center, Bar-Ilan UniversityRamat-Gan, Israel

**Keywords:** visual processing, object recognition, predictions, competition suppression, negative priming, top-down, ambiguity resolution

## Abstract

A substantial portion of information flow in the brain is directed top-down, from high processing areas downwards. Signals of this sort are regarded as conveying prior expectations, biasing the processing and eventual perception of incoming stimuli. In this perspective we describe a framework of top-down processing in the visual system in which predictions on the identity of objects in sight aid in their recognition. Focus is placed, in particular, on a relatively uncharted ramification of this framework, that of the fate of initial predictions that are eventually rejected during the process of selection. We propose that such predictions are rapidly inhibited in the brain after a competing option has been selected. Empirical support, along with behavioral, neuronal and computational aspects of this proposal are discussed, and future directions for related research are offered.

## Introduction

The hierarchical nature of information processing in the brain, particularly that of the visual system, has long been acknowledged. Originally, work focused on the accumulating complexity and sophistication added by each level of the hierarchy to the one preceding it, as information propagates upstream (Hubel and Wiesel, 1962). In recent years, however, a growing body of research has established the opposite direction of processing as well, from higher cortical areas downwards.

Top-down influences on perception are ubiquitous. From context (Biederman et al., 1982) and mood (Basso et al., 1996), to esthetic preference (Chen and Scholl, 2014) and inherent perceptual biases (Ramachandran, 1988), numerous factors intermix and contribute in shaping the subjective percept of a single objective stimulus. Many of these influences may be regarded as “predictions”, in the sense that they express prior expectations concerning the incoming stimulus. In Bar (2003), such a framework for top-down visual processing was offered, in which rapid implicit predictions are formed and utilized to facilitate object recognition. In this paper we shall first present this framework, and then discuss one aspect of it in particular—the fate of alternative, competing predictions that are not chosen.

## A framework for top-down facilitation of visual processing

Visual information is distributed across a range of spatial frequencies. Low spatial frequency (LSF) information carries gross outlines and object contours, while information of high spatial frequency (HSF) encodes edges and finer details of an image. LSFs of visual stimuli have been found to elicit early synchronized activity between primary visual areas and the orbitofrontal cortex (OFC), followed by a synchronized coupling of the OFC and object recognition regions of the inferior temporal (IT) cortex (Bar et al., 2006). This pattern of activation therefore seems to bypass the ventral bottom-up visual processing chain in reaching the prefrontal cortex (PFC), affording it early access to coarse general aspects of visual stimuli. It has been offered that this bypass pathway is enabled by magnocellular projections (Bar et al., 2006), which are quicker conductors and more attuned to LSF information compared with their parvocellular counterparts (Tootell et al., 1988; Merigan and Maunsell, 1993). Indeed, stimuli biasing magnocellular processing preferably activate the OFC and evoke a pattern of functional coupling between visual, OFC and IT regions (Kveraga et al., 2007). Robust afferent projections connect early visual areas and the OFC, both directly and indirectly (Barbas, 1995; Fuster, 2008), and several possible routes have been proposed to account for the rapid flow of activation in this pathway (Kveraga et al., 2007).

**Figure 1:**
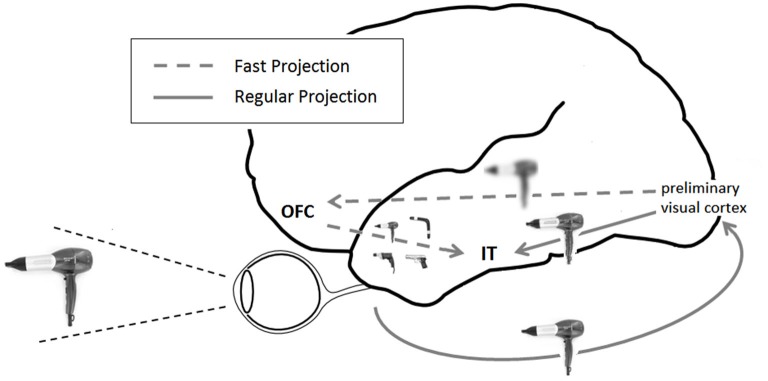
**Framework for top-down visual processing**. LSF information is rapidly projected from preliminary occipital visual areas to the OFC. Based on this rudimentary display, predictions are formed and projected downwards to IT regions, where they coincide with the slower ventral visual stream of processing and facilitate recognition. In this illustration, an image of hairdryer supposedly prompts predictions of a drill, a gun, a hairdryer and a boomerang.

According to the proposed framework, the OFC uses rudimentary versions of an input to rapidly activate familiar object categories resembling it. These activations act as hypotheses of the input’s identity, subsequently combined with HSF bottom-up processing in temporal regions to facilitate recognition (Bar et al., 2006; Figure 1). LSF-based “initial guesses” assist, therefore, in narrowing down the search space of identity matching. An image of a tennis racket, by this account, will evoke a cursory image in the OFC, which will cause a signal corresponding to a racket, a guitar, a spoon and other object prototypes sharing a relatively similar outline, to be subsequently passed downwards. Finding the best match between these options and additional bottom-up incoming information will constitute recognition. Combining bottom-up with top-down signals in this manner has indeed been demonstrated to yield optimal efficiency in a computational model of visual recognition (Graboi and Lisman, 2003). Bottom-up information confines the hypothesis space to a selected subset of options, which are then passed back downwards to subsequently confine the breadth of lower-level processing required.

Due to its cursory nature, LSF information is highly suitable for forming visual predictions of the sort described above. Hypotheses derived from it may be sufficiently guiding and at the same time not too specific or constrained. Such LSF-based hypotheses may be seen as object categories in that a blurred representation of an object typically encapsulates most of its category members. Indeed, LSF information facilitates object recognition that is flexible and resilient to changes in exemplars and viewpoints (Cheung and Bar, 2014). In this light, all LSF-based images are at least somewhat ambiguous. However, although some outlines are more informative and distinguishable than others (consider, for example, the outline of a bicycle), most LSF-based images would be expected to resemble and therefore prime not only different versions of the same object, but more than one object as well. A recognition process using LSF-based predictions foretells substantial constant ambiguity dealt with in the visual system. In this perspective we would like to extend our top-down visual processing framework by addressing a relatively overlooked ramification of it: the fate of the initial guesses that first compete but are eventually not chosen.

## Passive decay or active suppression

LSFs of objects in our visual environment typically evoke multiple possible interpretations of their identity. One of these guesses will ultimately be selected as the correct one as more details are conveyed by the HSFs and combined in analysis to constitute recognition. But what happens to the other alternative representations that have also been activated? Do these obsolete hypotheses gradually decay and die-out, neglected in the background while processing is concentrated elsewhere? Or is there rather active effort exerted for suppressing them rapidly? This question may provide promising new testing grounds for studying the strategy employed in the brain for conscious perception. Although no compelling evidence exists so far to support either possible account, in this perspective we would like to advocate as our hypothesis the latter, perhaps less intuitive, option of active suppression based on functional considerations.

At first glance, a mechanism in the brain for actively extinguishing activity that has been found irrelevant seems wasteful. Being irrelevant, such activity should lack encouragement and autonomously decay, so there is no apparent need in investing additional energy in putting it out quickly. In the next section we shall describe why we believe this mechanism is plausible and in fact necessary in the brain, by considering the possible benefits it might hold from a system point of view. Next, we shall support our claim by describing various findings of such a strategy employed in the brain in other domains. In the last section of this perspective we shall put forward our prediction regarding the mechanism of visual initial-guess competition suppression, and its testable manifestations.

## Benefits of suppressing un-chosen predictions

Visual predictions as suggested by our framework are helpful in parsing a visual scene. However, once one of the predictions had been chosen, the remaining alternatives become a potential interference to network dynamics. Unless quickly extinguished, such activations may, presumably, distort processing of new information in light of some ambiguity that had already been settled. Their efficient and abrupt dismissal seems therefore important for ongoing performance. This is potentially true regarding any predictive system in the brain. In the case of visual recognition, however, chosen predictions become conscious perceptions, and so the potential threat of distracting activations may be most pronounced.

An elementary characteristic of conscious experience is its unequivocal explicitness. Despite the bombardment of information we are constantly confronted with, which is both noisy and partial, our personal sensation is typically unitary, coherent and unambiguous. This is rather striking considering the breadth of subliminal activity we know is evoked and processed simultaneously in the brain at any given time. Indeed, consciousness is portrayed in many cases as an all-or-nothing mechanism, compared with subliminal workings that are statistical and inconclusive (Charles et al., 2013). To achieve this task, the nervous system must therefore be quick and decisive regarding interpretation of the world, selecting only a single percept to dominate consciousness at any given time. To this end, inhibition of the un-chosen options seems cardinal.

Current theories portray consciousness as the product of a large-scale connected component in neural network dynamics (Dehaene et al., 2003; Dehaene and Changeux, 2011). A mechanism for focusing activity on one selected option by suppressing irrelevant surroundings is important for the stability of such a component. Strong surrounding activations could otherwise potentially sway dynamics, even to the point of joining the locus of activation, which would result in a different conscious percept according to this theory. Therefore, efficient inhibition should take effect, and materialize rather immediately after selection when competition is highest and stability most fragile. This would be crucial for keeping our perception coherent. An extreme illustration of this idea may be found in the case of binocular rivalry, where strong and evenly matched competing activations are artificially created. The result is a non-stable perception alternating between the two of them (Blake and Logothetis, 2002).

The mechanism we propose in this perspective, therefore, is one aimed at “conscious decisiveness” and not ambiguity resolution *per se*. In our opinion, inhibition does not accentuate a single option over all others and thus helps in choosing between them, but rather it swiftly removes activations once such a choice has been made. The role ascribed to it is in “protecting” an implicit decision from interference once one is taken, by teasing out the to-be-explicit from the pool of implicit activations as quickly as possible to eliminate competition. Our argument is therefore for a post-selection and not a pre-selection mechanism, in agreement with most findings of competition suppression in the brain (May et al., 1995). In the next section we will overview several such findings, describing existing empirical evidence that provide initial support to our proposal.

## Evidence of active competition suppression

In the realm of linguistics, ambiguity resolution has drawn considerable interest over the years, and has been studied as a cognitive phenomenon to the greatest extent. It therefore provides good grounds from which to build our argument dealing with LSF-based ambiguity resolution.

### Suppression of competing interpretations

Numerous studies have shown that when an ambiguous word is encountered, several possible meanings of that word are immediately primed (Simpson, 1994), but access to all but the correct meaning of the word is significantly degraded within under a second of stimulus onset (Seidenberg et al., 1982; Gernsbacher and Faust, 1991b). Such decline in activation seems neither due to natural signal decay nor to competitive mutual inhibition between concepts (Gernsbacher and Faust, 1991b). Access to incorrect meanings may even become slower than access to neutral meanings that were not activated to begin with, providing further challenge to a mere decay account. This was shown in a lexical ambiguity experiment in which stimuli did not repeat, suggesting it is also not due to a prevalent memory-based explanation of such phenomena (Nievas and Marí-Beffa, 2002). It is interesting to note that the extent of suppression had been found in that study to be modulated by the strategy employed by subjects. Mirroring similar findings from selective attention (Neill and Westberry, 1987), suppression was exerted mostly when emphasis was placed on accuracy over speed, possibly indicating that it is mostly recruited when distractors are most harmful (Nievas and Marí-Beffa, 2002). Active inhibition, in any case, seems to take part in suppression of ambiguous word meanings, and its effect develops within a certain limited delay of selection.

Suppression in a linguistic task had been found when response was probed close to, but not immediately after, ambiguous primes. This was tested and shown as close as 467 ms from prime onset (Gernsbacher and Faust, 1991b), and analogous effects were also found under other modalities and tasks, within 1 s or less (Gernsbacher and Faust, 1991a). In one visual experiment, for example, subjects were presented with arrays of objects on screen for 250 ms, and then later shown a target and asked whether it was present in that array or not (Gernsbacher and Faust, 1991a). As expected, it was found that when targets were not part of the preceding array it took longer to reject them if they were contextually related to the array than if they were not (following an array of farm-related objects it took longer to reject the target “tractor” than it did to reject the target “kettle”). An interesting difference, however, emerged within subjects between “less-skilled comprehenders” and “more-skilled comprehenders” when targets were shown not immediately after the array display, but 1 s later. After a 1 s delay, the prolonged response times incurred by contextual relatedness remained in less-skilled comprehenders, while they had completely disappeared in skilled comprehenders. Successful comprehension of a visual scene seems to rely to some extent on the efficient suppression of potential distractors. This paradigm has proven so fruitful that suppression of inappropriate options was argued to be an overarching pivotal skill in comprehension in general (Gernsbacher and Faust, 1991a).

### Subliminally induced competition

In the studies discussed above, competing alternatives stemmed from conscious perception of an ambiguous stimulus. Their suppression may therefore be seen as a form of cognitive inhibition, known to be a major factor in a wide array of decision-making tasks, supposedly controlled by executive functions in the PFC (Miyake et al., 2000; Aron et al., 2004). LSF of visual objects, however, are presumed to elicit a fleeting subliminal perception. Here we present findings from motor control research that support our proposal of predictive activation and subsequent inhibition that are triggered subliminally.

In Eimer and Schlaghecken (1998), subjects were to press either a left or a right key, as fast and accurately as possible, according to given cues. Each cue was preceded by an additional masked prime cue that was not consciously perceived. It was found that primes harmed performance when the subsequent task was congruent with them, and on the other hand improved performance when the responses associated with the prime and the task were incongruent. Researchers called this phenomenon the “negative compatibility effect” (NCE), and offered that it is a result of inhibition acting on responses which were only partially activated (Eimer and Schlaghecken, 1998, 2003). A key insight from this study is that performance in this type of task depends not only on prime-target compatibility, but also on their relative timing. Event-related potential recordings of motor cortex during this task revealed that primes elicit activation corresponding with the movement they denote within roughly 200 ms of their presentation, but this activity reverses polarity 100 ms later and dwells below baseline for around 100 ms (between 300–400 ms of prime onset). The researchers found that when a motor choice is made in this latter time frame, performance is impaired if a compatible cue was given before the target, and conversely enhanced by a “misleading cue”. If timed right, the primed alternative will in fact be suppressed beneath threshold.

This effect had been replicated and proven rather robust in the motor system. Importantly, when varying time intervals between primes and targets, NCE was only observed following longer delays (96–192 ms), while short delays of up to 32 ms generated positive priming, in compliance with a rise-and-fall activation pattern (Schlaghecken and Eimer, 2000). Eimer and Schlaghecken (2003) propose stimulus-driven inhibition as a faculty complimentary to conscious cognitive control. They call it “exogenous” inhibition, in contrast to the conscious cognitive inhibition of responses, which is “endogenous” (Eimer and Schlaghecken, 2003). Competition suppression of the NCE phenomenon validates the possibility of competing elements subliminally activated and then inhibited in brain functioning, and demonstrates such inhibition acting fast (within 100 ms).

## Experimental predictions of initial-guess suppression

Building on the two paradigms described above, the supporting evidence for multiple-alternative activation and suppression in ambiguity resolution, and the possible evocation of them subliminally, we now turn to describe the testable manifestations we expect suppression of visual initial-guesses will have.

### Time frame

Active suppression of a concept in memory will cause an increased difficulty in accessing that concept for a certain amount of time thereafter. This is termed negative priming, as it is the exact opposite of classic positive priming in which recently activated concepts enjoy advantageous processing. Initial-guess suppression, as we propose, should therefore join the varied multitude of cognitive tasks that behaviorally manifest as negative priming for a certain amount of time. Because the speed of visual object recognition depends on numerous aspects of the stimulus (image complexity, display duration, contextual information, familiarity, and so on) we align the temporal description of our model to the time point of recognition.

**Figure 2:**
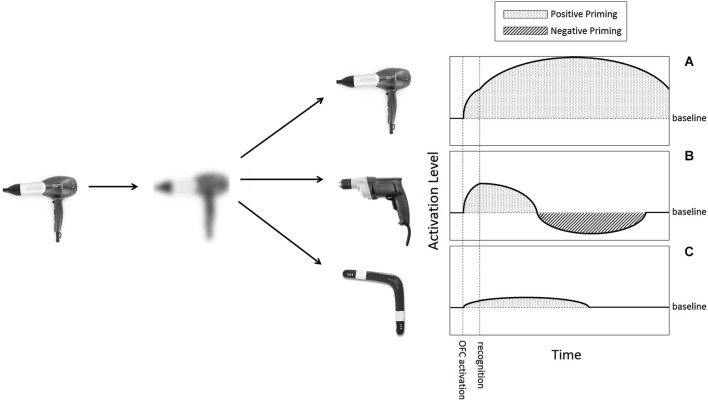
**Proposed activation patterns of LSF-induced hypotheses**. When presented an object (a hairdryer in this example), an LSF version of it is extracted and projected to the OFC. This cursory image activates resembling objects in memory, acting as hypotheses for recognition. In this case three such hypotheses are depicted.** (A)** The correct hypothesis is activated when an image reaches the OFC, like any other hypothesis. When additional information combines with it for recognition, it receives additional encouragment and will therefore display a classic positive priming affect for substantial time. **(B)** Competing initial hypotheses (a drill in this example), will initially be activated, but once recognition of a competing option will be made, our model proposes they will go under inhibition. Activation of an obsolete hypothesis of this sort should begin dropping and cross baseline activity. Undershooting the baseline will manifest as negative priming for a certain short period of time. **(C)** An example of an especially weak hypotheses, bearning minimal resemblance to the original image. According to several findings of threshold-dependent behavior of inhibition, a weak activation of this sort may not be inhibited at all, despite it being an un-chosen hypothesis like **(B)**.

We expect concepts activated by visual LSF information to be subsequently suppressed when additional information arrives and confirms one of the competing hypotheses. Visual LSF information creates enhanced activity in OFC areas around 50 ms before major activation of object-recognition areas of the IT begins (peaking at 130 and 180 ms, respectively, in the paradigm used in Bar et al. (2006)). This is the time window LSF-based generation of initial guesses happens, according to our proposition, so at this time interval multiple concepts should be activated and positively primed (Figure 2, between “OFC activation” and “recognition”).

Next, following strong coupling between the OFC and IT regions, recognition is achieved. It is this moment, or slightly earlier, that deems all but one activated guesses irrelevant, and so presumably ignites inhibition. Based on findings from the linguistic domain and others, we expect activation levels to drop below baseline level within less than 500 ms of this moment. The duration in which excitation should persist below baseline is hard to estimate. Various different negative priming paradigms have found an effect lasting between 0 and 8 s (May et al., 1995). Persistence would be particularly interesting to examine in the domain of vision, because visual context is typically rather stable over time, and so a mechanism for longer lasting suppression of irrelevant guesses might be desirable. A significant negative level of activation, in any case, is reasonable to expect for at least 100 ms (see Figure 2B for a summary of the predicted activation timeline of an un-chosen initial-guess).

### Inhibitory mechanism

Inhibition of un-chosen initial guesses, as hypothesized in this perspective, would operate only on the highest mode of visual processing and not trickle down to earlier regions of the visual pathway. Since selection is made between visually similar concepts (sharing major LSF features) activity patterns corresponding to them in low-level visual regions would be more similar than not. Inhibiting a low-level region for one concept and not the other would therefore be difficult, but mainly negligible. Behaviorally, as stated earlier, inhibition would manifest as negative priming between LSF-similar representations. However, alternative interpretations of such a finding exist and would have to be accounted for.

Whether negative priming indicates underlying inhibition has been the subject of a long and rich debate (May et al., 1995), but modern accounts tend to agree that both forward-acting inhibition and backward-acting memory mechanisms may give rise to negative priming under different experimental settings (Tipper, 2001; Mayr and Buchner, 2007). According to memory-based accounts, negative priming occurs when a task evokes previous processing episodes from memory and these episodes conflict with current settings. The conflict may lay in the response associated with the target of the task (previously ignored but currently requiring a response) (Neill et al., 1992) or in the different features the target object had in both episodes (Park and Kanwisher, 1994). A prerequisite for these accounts, in either case, is that the target serves as a good retrieval cue to a previous prime. This would seem less probable in an experiment examining suppression of LSF-based predictions. Unlike most negative priming paradigms, primes and targets of our framework would be dissimilar (a guitar and a tennis racket, for example). The supposed activation and rejection of the target in the prime episode, moreover, is implicit and not encoded in memory. Therefore, under experimental settings in which targets are fully visible and processed, it is unlikely that a reliance on memory retrieval would be promoted (May et al., 1995), and negative priming in these cases would be better explained by neuronal inhibition. Settings examining our framework would therefore favor an inhibition explanation, in our opinion, but careful design would nevertheless have to regard the alternative accounts.

Lastly, an additional research path, considering associative strength, may shed light on the characteristics of the inhibition we propose. In several studies, neural inhibition behaves in a seemingly threshold-dependent manner. In such cases, surprisingly no inhibition is applied when activation strength is particularly low. It has been found that activations that are especially weak are allowed to linger, spared the neural inhibition their counterparts receive, as if going “beneath the radar” of neural inhibition (Eimer and Schlaghecken, 2003; Tsushima et al., 2006). This aspect allows postulating that certain guesses may not be inhibited if their activation was particularly small to begin with. In our case, activations would be weak if the outline of the display and the outline of the guess are only remotely similar, analogous to a guess that is less probable (Figure 2C). This could be an interesting research path to follow, one that could yield firm evidence that the processes supporting this type of inhibition are similar to the ones supporting resembling phenomena mentioned here from selective attention and motor control.

## Summary

In this perspective we have overviewed our framework for top-down processing in the visual system, and focused on a particularly intriguing and understudied implication of it: the fate of un-chosen predictions. Albeit counter-intuitive, we believe that irrelevant activations of this sort undergo active inhibition in the brain. Such conduct seems important for maintaining stability and coherence in ongoing conscious perception. Reviewing evidence from different research areas of neuroscience, it seems that activation of numerous options, followed by coincided facilitation of one and inhibition of the rest, is characteristic of normal brain functioning. We hope future research will build on this proposal and elaborate it, as we believe further scientific study of this phenomenon will improve our understanding of efficient strategies for visual and non-visual information processing, and of human perception in general.

## Conflict of interest statement

The authors declare that the research was conducted in the absence of any commercial or financial relationships that could be construed as a potential conflict of interest.
